# Giant gastrointestinal stromal tumor with predominantly cystic changes: a case report and literature review

**DOI:** 10.1186/s12957-017-1285-2

**Published:** 2017-12-13

**Authors:** Liying Wang, Libin Liu, Zhaohui Liu, Yueli Tian, Zhe Lin

**Affiliations:** 1 Department of Gastroenterology, The Second Hospital of Jilin University, No. 218, Ziqiang Street, Changchun, 130042 China; 2 Department of Gastrointestinal Nutrition and Hernia Surgery, The Second Hospital of Jilin University, Changchun, China

**Keywords:** Gastrointestinal stromal tumor, Stomach, Cyst, Diagnosis

## Abstract

**Background:**

Gastrointestinal stromal tumors (GISTs) rarely present with predominantly cystic changes. Here, we report a case of giant GIST of the stomach with predominantly cystic changes in a 74-year-old female patient.

**Case presentation:**

The tumor was 10 cm × 15 cm in size and positive for CD117, *H*-caldesmon, and DOG-1. Complete surgical resection was performed without regional lymphadenectomy. The patient recovered uneventfully and no recurrence occurred.

**Conclusions:**

The case illustrates that GIST with cystic changes should be considered in the diagnoses of hepatic and pancreatic lesions. Furthermore, immunohistochemistry with CD117, DOG1, and other molecular markers is critical for diagnosis of GIST of the stomach and facilitates optimization of treatments for GIST.

## Background

Gastrointestinal stromal tumor (GIST) is the most common mesenchymal tumor of the digestive tract. Although GISTs only occur in less than 5% of all gastrointestinal tract tumors, they account for 60% of all gastric stromal tumors [1]. GISTs are typically solid tumors and rarely present with predominantly cystic changes [2]. Here, we describe a case of giant GIST of the stomach with predominantly cystic changes that was cured with complete surgical resection without regional lymphadenectomy.

## Case presentation

A 74-year-old female patient was hospitalized because of worsening abdominal pain for 2 days. The patient had distending upper abdominal pain for 3 months. The pain was intermittent and dull and did not radiate. She had no nausea or vomiting. Two days before admission, the pain worsened without apparent causes. The patient had no fever, and urine and stool were normal. She had no respiratory symptoms except occasional cough and whitish sputum. The patient had a history of hypertension for 5 years; the blood pressure was as high as 190/90 mmHg, and the patient took oral nifedipine (30 mg/day, sustained release tablet, Bayer).

**Fig. 1:**
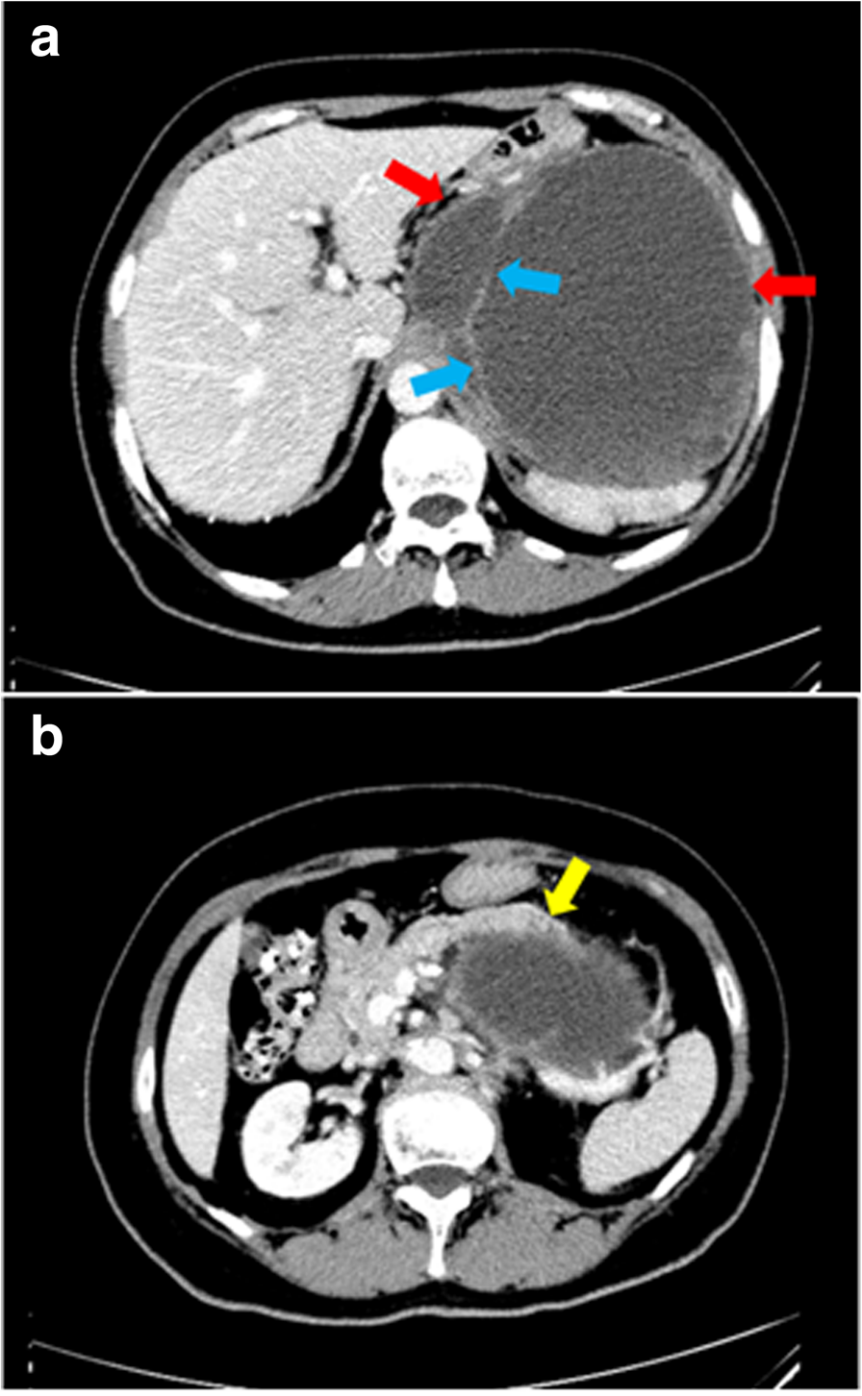
Abdominal CT demonstrates a cystic hypointense shadow in the upper left abdomen. **a** Septations (blue arrow) are seen in the tumor (red arrow), which is approximately 15.6 cm × 14.7 cm × 13.6 cm in size. **b** The pancreatic gland (yellow arrow) is compressed and displaced anteriorly, and the border between the pancreatic tail (white arrow) and the tumor is indistinct

Physical examination at admission showed tenderness in the mid and upper left abdomen, but no rebound tenderness was elicited. A mass was palpated in the upper left abdomen. The mass was approximately 10 cm in diameter and had an indistinct boundary and was poorly mobile. The findings were otherwise unremarkable. Laboratory findings including blood chemistries, routine blood tests, coagulation profile, and tumor markers were normal. Immunological tests for antigens or antibodies related to infections with *Treponema pallidum*, HIV (human immunodeficiency virus), and HCV (hepatitis C virus) were negative. Abdominal ultrasonography revealed a hypoechoic intraperitoneal mass in the upper left quadrant, 13.3 cm × 12.5 cm in size, with punctate hyperechoic deposits. The wall of the cyst was rough, with many irregular hyperechoic protuberances. Abdominal CT showed a cystic hypointense shadow with septations, approximately 15.6 cm × 14.7 cm × 13.6 cm in size (Fig. 1). The stomach was compressed by the tumor and was not clearly visualized. The pancreatic gland was displaced anteriorly by the tumor, and the border between the tail of the pancreas and the tumor was indistinct. The spleen and the left kidney were displaced inferiorly by the tumor. Gastric endoscopy revealed protuberances in the fundus, suggestive of external compression.

**Fig. 2:**
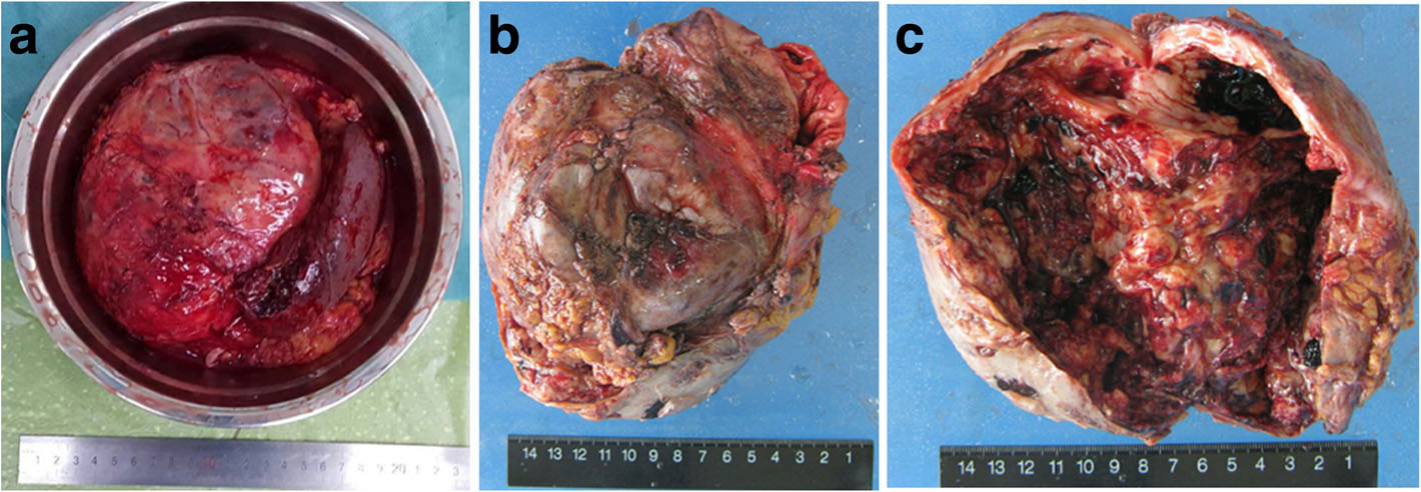
**a** The gross appearance of the excised tumor, which is 16 cm × 15 cm × 13 cm in size. The resected tumor is well circumscribed and gray-whitish. **b** The macroscopic aspect of the resected tumor with focal areas of necrosis and cystic degeneration. **c** The irregular wall of the cyst

Exploratory laparotomy showed a cystic mass in the left upper quadrant, 10 cm × 15 cm in size, starting from the posterior wall of the gastric fundus and had intimate relations with the spleen and the greater curvature of the gastric fundus. Rapid pathological examination indicated gastric stromal tumors. The tumor, the proximal stomach, and the spleen were excised. The tumor had a maximal diameter of 15 cm, with necrosis and cystic degeneration (Fig. 2). No invasion of the spleen was seen. No intraperitoneal lymph node metastasis (0/3) and no metastasis to peritumoral lymph nodes (0/6) were observed. Distant metastases, such as in the liver and lung, were also not observed. Postoperative pathological examination showed GIST (high grade, spindle cell variant). The mitotic index was 10/50 in high-power field. Immunohistochemical study revealed CD117 (+), H-caldesmon (+), SMA (weak +), DOG-1 (+), CD34 (−), desmin (−), Ki67 (5% positive), NSE (−), and S-100 (−). No peritoneal metastasis was present. The patient recovered uneventfully and discharged from hospital after 2 weeks. The patient received regular follow-up, and no recurrence has occurred at the time of writing this case report (20 months following surgery).

## Discussion and conclusions

GISTs are the most common mesenchymal tumors and account for 0.1 to 3% of all gastrointestinal tumors [1]. GISTs had been considered to be smooth muscle neoplasms such as leiomyoma or leiomyosarcoma; subsequent studies have demonstrated that GIST arises from c-KIT (CD117)-positive intestinal cells of Cajal in the gastrointestinal tract. The clinical manifestations of GIST are non-specific and vary from being asymptomatic to abdominal pain, a palpable mass, bleeding, intestinal occlusion, and perforation. The diagnosis of GIST of the stomach is frequently not clear preoperatively, as in the current case. GISTs of the stomach with cystic changes are not common and may be misdiagnosed as hepatic or pancreatic lesions [3, 4]. The tumor in our case had intimate relations with adjacent structures such as the liver and spleen and its border was indistinct from the pancreatic tail. Though abdominal ultrasonography, CT scan, and gastric endoscopy were performed in our patient, these diagnostic modalities failed to yield a definite diagnosis. Histologically, the tumor is composed of interlacing bundles or whorls of spindle-shaped cells. However, histological findings are noncommittal and definite diagnosis relies on immunohistochemical studies. CD117 is positive in 90% of the cases of GIST. The tumor tissue specimen in the current case was also intensely positive for CD117. Our patient was also positive for DOG1, another sensitive marker that is positive in 95% of the cases [4], but negative for CD34, which is positive in 50 to 80% of the cases [4].

One noticeable feature of our case is the presence of predominantly cystic changes in the tumor. GISTs often form solid tumors and are rarely predominantly cystic. GISTs with cystic changes are more frequently seen in high-grade malignancies. Because of aggressive tumor growth, necrosis, hemorrhage, liquefaction, and cystic degeneration occur from lack of adequate blood supply, leading to formation of large cystic spaces [5]. Necrosis and cystic degeneration were also seen in the tumor of our patient. GISTs are typically large tumors, with diameters greater than 10 cm. Okano et al. reviewed six reported cases of GIST with cystic changes, and the size of these tumors varied from 6 to 32 cm [2]. We further reviewed the English and Chinese literature and summarize the reported cases in Table 1 [4, 6–13]. These patients had a median age of 66 years (range 11 to 80 years) and five of them (45.5%, 5/11) were female. The cyst size ranged from 6 to 37.6 cm.

**Table 1:** Clinical features of reported cases of GISTs of the stomach with cystic formation in the literature

Case no.	Reference	Age, years	Gender	Size, cm	Location	Rupture or perforation	Mitotic figures	Surgery	Imatinib	Follow-up duration, months	Recurrence
1	Park et al.	11	F	10	The anterior gastric wall	No	–	Yes	Yes	9	No
2	Osada et al.	74	M	12	The gastric wall in the posterior aspect of the fundus	Yes	High mitotic count	Yes	Yes	24	No
3	Cruz Jr. et al.	37	M	32	The distal part of stomach	No	10/50 HPF	Yes	Yes	12	No
4	Yu et al.	80	F	6	The front of the stomach	Yes	4/50 HPF	Yes	–	–	–
5	Notani et al.	58	M	33	The gastric wall	No	–	Yes	Yes	12	No
6	Zhu et al.	78	M	17	The gastric wall	No	> 10/50 HPF	Yes	No	36	No
7	Okano et al.	79	M	60	The gastric wall	No	> 5/50 HPF	Yes	No	12	No
8	Colović et al.	52	F		The greater curvature of the stomach	No	–	Yes	Yes	24	No
9	Chen XQ et al.	60	M	28.5	The posterior wall of the gastric body	No	–	Yes	No	12	No
10	Yang CB	66	F	37.6	The posterior wall of the gastric body	No	–	Yes	–	–	–
11	The current case	74	F	15	The gastric wall in the posterior aspect of the fundus	No	> 10/50 HPF	Yes	No	20	No

GISTs possess malignant potential and are highly invasive and tend to metastasize to remote organs [8]. Metastases of GIST commonly develop in the abdominal cavity and liver, and rarely in the lymph nodes. Though necrosis and cystic degeneration were observed in the tumor specimen of our patient, we observed no metastasis to lymph nodes and remote organs. The tumor was completely encapsulated, and invasion to adjacent tissues was not seen. This may explain that no tumor recurrence has occurred following complete surgical resection of the tumor. Wang et al. reported seven cases of GIST with cystic changes and found no recurrence 9 to 80 months following surgical resection [14].

GISTs of the stomach with cystic changes are rare and may defy diagnosis preoperatively. Our case illustrates that GIST with cystic changes should be considered in the diagnoses of hepatic and pancreatic lesions. Furthermore, immunohistochemistry with CD117, DOG1, and other molecular markers is critical for diagnosis of GIST of the stomach and facilitates optimization of treatments for GIST.

## Abbreviations

GISTs: Gastrointestinal stromal tumors
HCV: Hepatitis C virus
HIV: Human immunodeficiency virus
